# (*E*)-1-(4-Nitro­phen­yl)-2-(4-{[(*E*)-2-(4-nitro­phen­yl)hydrazinyl­idene]meth­yl}benzyl­idene)hydrazine dihydrate

**DOI:** 10.1107/S1600536809053963

**Published:** 2009-12-19

**Authors:** Edward R. T. Tiekink, James L. Wardell, Solange M. S. V. Wardell

**Affiliations:** a Department of Chemistry, University of Malaya, 50603 Kuala Lumpur, Malaysia; b Centro de Desenvolvimento Tecnológico em Saúde (CDTS), Fundação Oswaldo Cruz (FIOCRUZ), Casa Amarela, Campus de Manguinhos, Av. Brasil 4365, 21040-900 Rio de Janeiro, RJ, Brazil; c CHEMSOL, 1 Harcourt Road, Aberdeen AB15 5NY, Scotland

## Abstract

The 30 non-H atoms in title dihydrazine compound, C_20_H_16_N_6_O_4_·2H_2_O, are close to coplanar, the r.m.s. deviation for these atoms being 0.096 Å. The conformation about each of the C=N bonds is *E*, and the mol­ecule has non-crystallographic 2/*m* symmetry. The presence of O—H⋯O and N—H⋯O hydrogen bonding leads to a three-dimensional network in the crystal structure. A highly disordered solvent mol­ecule is present within a mol­ecular cavity defined by the organic and water mol­ecules. Its contribution to the electron density was removed from the observed data in the final cycles of refinement and the formula, molecular weight and density are given without taking into account the contribution of the solvent molecule.

## Related literature

For background to the structural chemistry of hydrazones, see: Baddeley *et al.* (2009 ▶); Ferguson *et al.* (2005 ▶); Glidewell *et al.* (2006 ▶); Low *et al.* (2006 ▶); Wardell *et al.* (2005 ▶, 2006 ▶). For the synthesis, see: Bengelsdorf (1958 ▶).
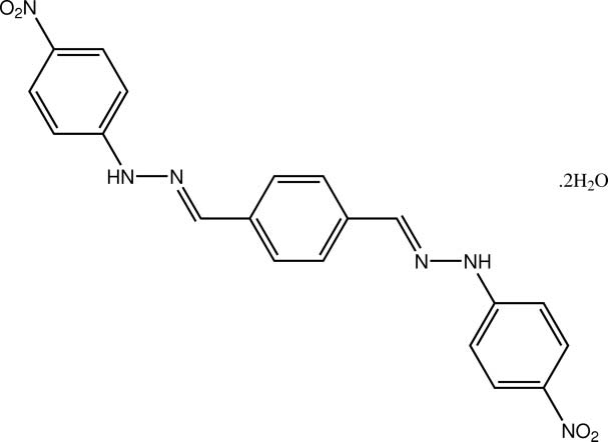


## Experimental

### 

#### Crystal data

C_20_H_16_N_6_O_4_·2H_2_O
*M* *_r_* = 440.42
Triclinic, 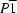

*a* = 7.7549 (4) Å
*b* = 9.3245 (7) Å
*c* = 15.3374 (11) Å
α = 100.749 (3)°
β = 90.533 (4)°
γ = 103.131 (5)°
*V* = 1059.56 (12) Å^3^
*Z* = 2
Mo *K*α radiation
μ = 0.11 mm^−1^
*T* = 120 K
0.38 × 0.22 × 0.07 mm

#### Data collection

Nonius KappaCCD area-detector diffractometer
Absorption correction: multi-scan (*SADABS*; Sheldrick, 2007 ▶) *T*_min_ = 0.770, *T*_max_ = 1.000
16566 measured reflections
3688 independent reflections
2638 reflections with *I* > 2σ(*I*)
*R*_int_ = 0.039

#### Refinement

*R*[*F* ^2^ > 2σ(*F* ^2^)] = 0.060
*wR*(*F* ^2^) = 0.177
*S* = 1.09
3688 reflections
301 parameters
6 restraints
H atoms treated by a mixture of independent and constrained refinement
Δρ_max_ = 0.45 e Å^−3^
Δρ_min_ = −0.30 e Å^−3^

### 

Data collection: *COLLECT* (Hooft, 1998 ▶); cell refinement: *DENZO* (Otwinowski & Minor, 1997 ▶) and *COLLECT*; data reduction: *DENZO* and *COLLECT*; program(s) used to solve structure: *SHELXS97* (Sheldrick, 2008 ▶); program(s) used to refine structure: *SHELXL97* (Sheldrick, 2008 ▶) and *PLATON* (Spek, 2009 ▶); molecular graphics: *ORTEP-3* (Farrugia, 1997 ▶) and *DIAMOND* (Brandenburg, 2006 ▶); software used to prepare material for publication: *publCIF* (Westrip, 2010 ▶).

## Supplementary Material

Crystal structure: contains datablocks global, I. DOI: 10.1107/S1600536809053963/hg2618sup1.cif

Structure factors: contains datablocks I. DOI: 10.1107/S1600536809053963/hg2618Isup2.hkl

Additional supplementary materials:  crystallographic information; 3D view; checkCIF report

## Figures and Tables

**Table 1:** Hydrogen-bond geometry (Å, °)

*D*—H⋯*A*	*D*—H	H⋯*A*	*D*⋯*A*	*D*—H⋯*A*
O1*W*—H1w⋯O4^i^	0.83 (2)	2.32 (2)	3.084 (2)	153 (2)
O1*W*—H2w⋯O2w^ii^	0.83 (2)	2.01 (2)	2.808 (3)	163 (3)
N5—H5n⋯O1w	0.88	2.17	3.021 (3)	163
O2*W*—H3w⋯O1w^iii^	0.84 (3)	1.98 (3)	2.800 (3)	165 (3)
O2*W*—H4w⋯O1^iv^	0.84 (2)	2.28 (2)	3.061 (3)	156 (3)
O2*W*—H4w⋯O2^iv^	0.84 (2)	2.45 (2)	3.204 (3)	150 (3)
N2—H2n⋯O2w^v^	0.88	2.09	2.959 (3)	172
C7—H7⋯O2^vi^	0.95	2.48	3.374 (3)	157
C14—H14⋯O3^i^	0.95	2.45	3.338 (3)	156

Symmetry codes: (i) 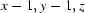; (ii) 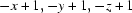; (iii) 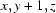; (iv) 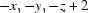; (v) 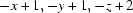; (vi) 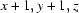.

**Fig. 1.**
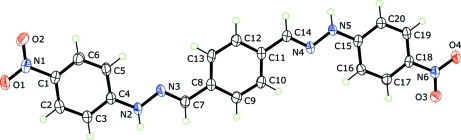
The molecular structure of (I) showing the atom-labelling scheme and displacement ellipsoids at the 50% probability level.

**Fig. 2.**
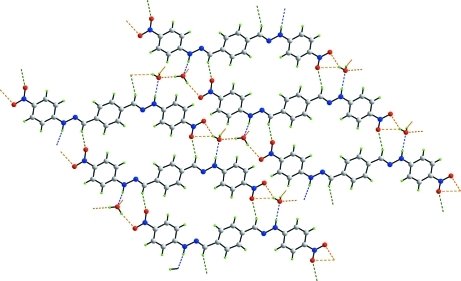
A view of the supramolecular 2-D array in (I) mediated by O–H···O and N–H···O hydrogen bonding shown as orange and blue dashed lines, respectively. Additional C–H···O contacts are shown as green dashed lines, Colour code: O, red; N, blue; C, grey; and H, green.

**Fig. 3.**
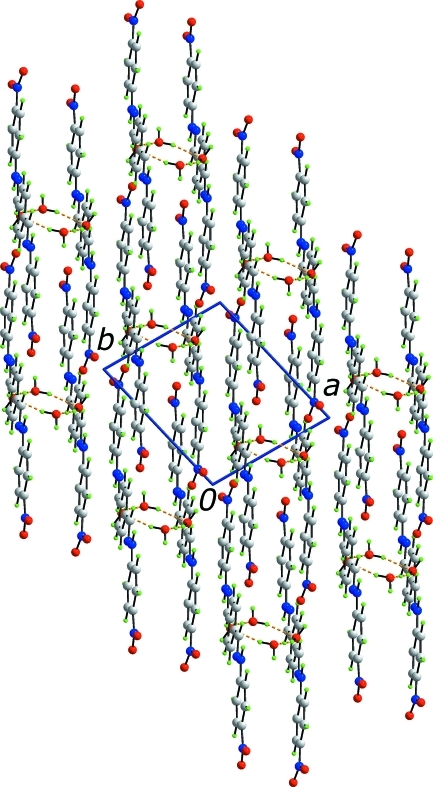
A view of the stacking of layers (illustrated in Fig. 2) in (I) with the O–H···O hydrogen bonding connecting the layers shown as orange dashed lines. Colour code: O, red; N, blue; C, grey; and H, green.

## supplementary crystallographic information

### Comment

In connection with on-going studies into the structural chemistry of hydrazones (Baddeley *et al.*, 2009; Ferguson *et al.*, 2005; Glidewell *et al.*, 2006; Low *et al.*, 2006; Wardell *et al.*, 2005; Wardell *et al.*, 2006), we now report the structure of the title compound, (I).

The molecule in (I) is essentially planar with the r.m.s. of the 30 non-hydrogen atoms being 0.096 Å. The maximum deviations from the least-squares plane are 0.243 (2) Å for atom O3 and -0.140 (3) Å for atom C13; the former deviation arises as the N6-nitro group is slightly twisted out of the plane of the benzene ring to which it is attached: the C17–C18–N6–O3 torsion angle is 7.1 (3)°. The conformation about each of the C7═ N3 [1.288 (3) Å] and C14═N4 [1.280 (3) Å] bonds is *E*. Overall, to a first approximation, the molecule has non-crystallographic 2/m symmetry.

The water molecules are involved in a number of hydrogen bonding interactions and stabilize a double layer arrangement. As illustrated in Fig. 2, molecules are arranged into a layer being connected by O–H···O and N–H···O hydrogen bonds as well as C–H···O contacts, Table 1. Each of the hydrazine-H atoms forms a donor interaction to a water molecule. The O1w water molecule forms a donor hydrogen bond with a O2w water molecule in the plane, Fig. 2, as well as with a nitro-O4 atom. The O2w water molecule accepts a hydrogen bond from the O1w atom as described above, and forms two donor interactions with the nitro-O1 and O2 atoms *via* a bifurcated H4w atom. Each of the nitro O1 and O2 atoms forms a C–H···O contact. The aforementioned interactions stabilize a 2-D array. Each of the O1w (acceptor) and O2w (donor) molecules forms one further hydrogen bond to a water molecule of a centrosymmetrically related layer to form a double layer as well as eight-membered {···O—H}_4_ synthons. Further stability to the double layers is afforded by weak π···π interactions [ring centroid(C16–C6)···ring centroid(C15—C20)^i^ = 3.6716 (16) Å with a dihedral angle between planes = 2.31 (12) ° for symmetry operation i: 1 - *x*, -*y*, 2 - *z*]. Layers stack in the crystal structure as illustrated in Fig. 3. As noted in the Experimental, ill-defined solvent, most probably methanol, was present in the crystal structure. These are located in the vicinity of the voids within the double layer.

### Experimental

Solutions of 4-nitrophenylhydrazine (0.306 g, 2 mmol) in MeOH (25 ml) and 1,4-benzenedicarboxaldehyde (0.134 g, 1 mmol) in MeOH (15 ml) were mixed, and refluxed for 30 min. The reaction mixture was rotary evaporated and the residue was chromatographed on alumina using hexane/ethyl acetate [4:1] as eluent. The collected fraction of 1,4-bis-2-(4-nitrophenyl)hydrazone 1,4-benzenedicarboxaldehyde was recrystallized from MeOH, m.pt. 566–568 K. lit value 567–568 K (Bengelsdorf, 1958). IR (KBr, cm^-1^): ν 3261, 1608, 1587, 1556, 1498, 1469, 1321, 1309, 1293, 1271, 1173, 1105, 1086, 998, 929, 839, 750, 694, 585, 530, 489, 435.

### Refinement

The N– and C-bound H atoms were geometrically placed (N–H = 0.88 Å and C–H = 0.95 Å) and refined as riding with *U_iso_*(H) = 1.2*U_eq_*(C). The O–H atoms were located in a difference map and refined with the distance restraint O–H = 0.84±0.01 and with *U_iso_*(H) = 1.5*U_eq_*(N). Unresolved disordered solvent was evident in the final cycles of the refinement. This was modelled with the SQUEEZE option in *PLATON* (Spek, 2009); the solvent cavity had volume 76 Å^3^. In the final cycles of refinement, this contribution to the electron density was removed from the observed data. The density, the F(000) value, the molecular weight, and the formula are given without taking into account the contribution of the solvent molecule.

### Crystal data

**Table:**

C_20_H_16_N_6_O_4_·2H_2_O	*Z* = 2
*M**_r_* = 440.42	*F*(000) = 460
Triclinic, *P*1	*D*_x_ = 1.380 Mg m^−^^3^
Hall symbol: -P 1	Mo *K*α radiation, λ = 0.71073 Å
*a* = 7.7549 (4) Å	Cell parameters from 4364 reflections
*b* = 9.3245 (7) Å	θ = 2.9–27.5°
*c* = 15.3374 (11) Å	µ = 0.11 mm^−^^1^
α = 100.749 (3)°	*T* = 120 K
β = 90.533 (4)°	Block, dark-red
γ = 103.131 (5)°	0.38 × 0.22 × 0.07 mm
*V* = 1059.56 (12) Å^3^	

### Data collection

**Table:**

Nonius KappaCCD area-detector diffractometer	3688 independent reflections
Radiation source: Enraf–Nonius FR591 rotating anode	2638 reflections with *I* > 2σ(*I*)
10 cm confocal mirrors	*R*_int_ = 0.039
Detector resolution: 9.091 pixels mm^-1^	θ_max_ = 25.0°, θ_min_ = 3.0°
φ and ω scans	*h* = −9→8
Absorption correction: multi-scan (*SADABS*; Sheldrick, 2007)	*k* = −11→11
*T*_min_ = 0.770, *T*_max_ = 1.000	*l* = −18→18
16566 measured reflections	

### Refinement

**Table:**

Refinement on *F*^2^	Primary atom site location: structure-invariant direct methods
Least-squares matrix: full	Secondary atom site location: difference Fourier map
*R*[*F*^2^ > 2σ(*F*^2^)] = 0.060	Hydrogen site location: inferred from neighbouring sites
*wR*(*F*^2^) = 0.177	H atoms treated by a mixture of independent and constrained refinement
*S* = 1.09	*w* = 1/[σ^2^(*F*_o_^2^) + (0.0852*P*)^2^ + 0.5693*P*] where *P* = (*F*_o_^2^ + 2*F*_c_^2^)/3
3688 reflections	(Δ/σ)_max_ < 0.001
301 parameters	Δρ_max_ = 0.45 e Å^−^^3^
6 restraints	Δρ_min_ = −0.30 e Å^−^^3^

### Special details

**Table:**

Geometry. All s.u.'s (except the s.u. in the dihedral angle between two l.s. planes) are estimated using the full covariance matrix. The cell s.u.'s are taken into account individually in the estimation of s.u.'s in distances, angles and torsion angles; correlations between s.u.'s in cell parameters are only used when they are defined by crystal symmetry. An approximate (isotropic) treatment of cell s.u.'s is used for estimating s.u.'s involving l.s. planes.
Refinement. Refinement of *F*^2^ against ALL reflections. The weighted *R*-factor *wR* and goodness of fit *S* are based on *F*^2^, conventional *R*-factors *R* are based on *F*, with *F* set to zero for negative *F*^2^. The threshold expression of *F*^2^ > 2σ(*F*^2^) is used only for calculating *R*-factors(gt) *etc*. and is not relevant to the choice of reflections for refinement. *R*-factors based on *F*^2^ are statistically about twice as large as those based on *F*, and *R*- factors based on ALL data will be even larger.

### Fractional atomic coordinates and isotropic or equivalent isotropic displacement parameters (Å^2^)

**Table:**

	*x*	*y*	*z*	*U*_iso_*/*U*_eq_	
O1	−0.2175 (2)	−0.6067 (2)	1.41897 (13)	0.0387 (5)	
O2	−0.3174 (3)	−0.6708 (2)	1.28198 (14)	0.0421 (5)	
O3	1.1204 (2)	0.9370 (2)	0.69699 (13)	0.0388 (5)	
O4	0.9881 (2)	0.8999 (2)	0.56744 (12)	0.0340 (5)	
N1	−0.2205 (3)	−0.5823 (2)	1.34244 (16)	0.0314 (5)	
N2	0.2344 (3)	−0.0749 (2)	1.26201 (14)	0.0285 (5)	
H2N	0.3112	−0.0149	1.3030	0.034*	
N3	0.2300 (3)	−0.0459 (2)	1.17772 (14)	0.0279 (5)	
N4	0.5188 (3)	0.3449 (2)	0.80714 (13)	0.0268 (5)	
N5	0.5234 (3)	0.3707 (2)	0.72248 (13)	0.0272 (5)	
H5N	0.4508	0.3089	0.6803	0.033*	
N6	1.0012 (3)	0.8658 (2)	0.64104 (14)	0.0281 (5)	
C1	−0.1068 (3)	−0.4476 (3)	1.32299 (17)	0.0264 (6)	
C2	0.0140 (3)	−0.3527 (3)	1.38838 (18)	0.0298 (6)	
H2	0.0190	−0.3740	1.4464	0.036*	
C3	0.1262 (3)	−0.2277 (3)	1.36776 (17)	0.0275 (6)	
H3	0.2084	−0.1618	1.4119	0.033*	
C4	0.1193 (3)	−0.1974 (3)	1.28162 (16)	0.0250 (6)	
C5	−0.0061 (3)	−0.2935 (3)	1.21764 (18)	0.0312 (6)	
H5	−0.0141	−0.2721	1.1597	0.037*	
C6	−0.1168 (4)	−0.4174 (3)	1.23830 (19)	0.0330 (6)	
H6	−0.2005	−0.4829	1.1946	0.040*	
C7	0.3492 (3)	0.0666 (3)	1.16298 (17)	0.0285 (6)	
H7	0.4329	0.1230	1.2094	0.034*	
C8	0.3571 (3)	0.1086 (3)	1.07560 (17)	0.0270 (6)	
C9	0.4848 (4)	0.2336 (3)	1.06298 (18)	0.0371 (7)	
H9	0.5628	0.2913	1.1114	0.044*	
C10	0.4993 (4)	0.2748 (3)	0.98095 (18)	0.0365 (7)	
H10	0.5857	0.3617	0.9740	0.044*	
C11	0.3902 (3)	0.1917 (3)	0.90894 (17)	0.0267 (6)	
C12	0.2590 (4)	0.0692 (3)	0.92239 (19)	0.0399 (7)	
H12	0.1792	0.0129	0.8743	0.048*	
C13	0.2436 (4)	0.0288 (3)	1.00415 (19)	0.0400 (7)	
H13	0.1535	−0.0554	1.0117	0.048*	
C14	0.4076 (3)	0.2275 (3)	0.82018 (17)	0.0274 (6)	
H14	0.3356	0.1628	0.7716	0.033*	
C15	0.6399 (3)	0.4935 (3)	0.70347 (16)	0.0246 (6)	
C16	0.7616 (3)	0.5904 (3)	0.76914 (16)	0.0259 (6)	
H16	0.7625	0.5721	0.8280	0.031*	
C17	0.8788 (3)	0.7112 (3)	0.74798 (17)	0.0267 (6)	
H17	0.9626	0.7757	0.7920	0.032*	
C18	0.8752 (3)	0.7394 (3)	0.66236 (17)	0.0251 (6)	
C19	0.7543 (3)	0.6461 (3)	0.59689 (17)	0.0275 (6)	
H19	0.7515	0.6670	0.5387	0.033*	
C20	0.6385 (3)	0.5231 (3)	0.61741 (17)	0.0274 (6)	
H20	0.5569	0.4577	0.5727	0.033*	
O1W	0.3412 (2)	0.1327 (2)	0.56735 (12)	0.0346 (5)	
H1W	0.2326 (16)	0.095 (3)	0.565 (2)	0.052*	
H2W	0.386 (4)	0.141 (4)	0.5191 (12)	0.052*	
O2W	0.4975 (2)	0.9015 (2)	0.59601 (13)	0.0349 (5)	
H3W	0.434 (3)	0.961 (3)	0.590 (2)	0.052*	
H4W	0.429 (3)	0.826 (2)	0.609 (2)	0.052*	

### Atomic displacement parameters (Å^2^)

**Table:**

	*U*^11^	*U*^22^	*U*^33^	*U*^12^	*U*^13^	*U*^23^
O1	0.0431 (11)	0.0390 (12)	0.0363 (12)	0.0049 (9)	0.0133 (9)	0.0185 (9)
O2	0.0404 (11)	0.0315 (11)	0.0495 (13)	−0.0048 (9)	−0.0040 (10)	0.0116 (10)
O3	0.0373 (11)	0.0373 (11)	0.0372 (12)	−0.0057 (9)	−0.0012 (9)	0.0132 (9)
O4	0.0418 (11)	0.0321 (11)	0.0311 (11)	0.0050 (8)	0.0072 (8)	0.0175 (8)
N1	0.0298 (12)	0.0267 (12)	0.0424 (15)	0.0093 (10)	0.0104 (10)	0.0146 (11)
N2	0.0320 (12)	0.0277 (12)	0.0272 (12)	0.0028 (9)	0.0048 (9)	0.0136 (9)
N3	0.0344 (12)	0.0279 (12)	0.0247 (12)	0.0085 (10)	0.0076 (9)	0.0115 (9)
N4	0.0301 (12)	0.0279 (12)	0.0259 (12)	0.0073 (9)	0.0064 (9)	0.0129 (9)
N5	0.0301 (12)	0.0277 (12)	0.0227 (12)	0.0000 (9)	0.0038 (9)	0.0106 (9)
N6	0.0319 (12)	0.0258 (12)	0.0296 (13)	0.0075 (10)	0.0078 (10)	0.0115 (10)
C1	0.0244 (13)	0.0230 (13)	0.0345 (15)	0.0062 (11)	0.0077 (11)	0.0110 (11)
C2	0.0325 (14)	0.0327 (15)	0.0309 (15)	0.0132 (12)	0.0116 (11)	0.0155 (12)
C3	0.0282 (14)	0.0284 (14)	0.0279 (14)	0.0059 (11)	0.0065 (11)	0.0108 (11)
C4	0.0262 (13)	0.0246 (13)	0.0278 (14)	0.0089 (10)	0.0078 (10)	0.0095 (11)
C5	0.0352 (15)	0.0308 (15)	0.0302 (15)	0.0045 (12)	0.0016 (12)	0.0161 (12)
C6	0.0336 (15)	0.0291 (15)	0.0368 (16)	0.0054 (12)	−0.0007 (12)	0.0102 (12)
C7	0.0289 (14)	0.0270 (14)	0.0300 (15)	0.0029 (11)	0.0040 (11)	0.0111 (11)
C8	0.0278 (14)	0.0264 (14)	0.0296 (14)	0.0070 (11)	0.0068 (11)	0.0115 (11)
C9	0.0369 (15)	0.0376 (16)	0.0303 (16)	−0.0083 (12)	−0.0002 (12)	0.0116 (12)
C10	0.0377 (16)	0.0336 (15)	0.0336 (16)	−0.0077 (12)	0.0036 (12)	0.0146 (13)
C11	0.0275 (13)	0.0264 (14)	0.0299 (15)	0.0074 (11)	0.0068 (11)	0.0132 (11)
C12	0.0414 (16)	0.0389 (17)	0.0334 (16)	−0.0101 (13)	−0.0032 (12)	0.0157 (13)
C13	0.0405 (16)	0.0368 (16)	0.0383 (17)	−0.0111 (13)	0.0025 (13)	0.0206 (13)
C14	0.0300 (14)	0.0240 (14)	0.0284 (14)	0.0030 (11)	0.0031 (11)	0.0095 (11)
C15	0.0257 (13)	0.0219 (13)	0.0287 (14)	0.0074 (10)	0.0074 (10)	0.0088 (11)
C16	0.0298 (14)	0.0286 (14)	0.0220 (13)	0.0084 (11)	0.0061 (10)	0.0097 (11)
C17	0.0283 (14)	0.0256 (14)	0.0264 (14)	0.0049 (11)	0.0033 (10)	0.0073 (11)
C18	0.0248 (13)	0.0227 (13)	0.0312 (15)	0.0066 (10)	0.0080 (11)	0.0121 (11)
C19	0.0327 (14)	0.0280 (14)	0.0259 (14)	0.0085 (11)	0.0073 (11)	0.0131 (11)
C20	0.0295 (14)	0.0263 (14)	0.0264 (14)	0.0035 (11)	0.0028 (11)	0.0090 (11)
O1W	0.0327 (11)	0.0375 (11)	0.0319 (11)	0.0007 (9)	0.0043 (8)	0.0118 (9)
O2W	0.0358 (11)	0.0304 (11)	0.0409 (12)	0.0022 (8)	0.0049 (9)	0.0192 (9)

### Geometric parameters (Å, °)

**Table:**

O1—N1	1.238 (3)	C8—C13	1.382 (4)
O2—N1	1.235 (3)	C8—C9	1.393 (4)
O3—N6	1.235 (3)	C9—C10	1.381 (4)
O4—N6	1.237 (3)	C9—H9	0.9500
N1—C1	1.444 (3)	C10—C11	1.379 (4)
N2—C4	1.364 (3)	C10—H10	0.9500
N2—N3	1.371 (3)	C11—C12	1.395 (4)
N2—H2N	0.8796	C11—C14	1.461 (3)
N3—C7	1.288 (3)	C12—C13	1.374 (4)
N4—C14	1.280 (3)	C12—H12	0.9500
N4—N5	1.364 (3)	C13—H13	0.9500
N5—C15	1.368 (3)	C14—H14	0.9500
N5—H5N	0.8799	C15—C20	1.398 (3)
N6—C18	1.442 (3)	C15—C16	1.406 (4)
C1—C6	1.384 (4)	C16—C17	1.370 (3)
C1—C2	1.393 (4)	C16—H16	0.9500
C2—C3	1.377 (3)	C17—C18	1.388 (3)
C2—H2	0.9500	C17—H17	0.9500
C3—C4	1.405 (3)	C18—C19	1.389 (4)
C3—H3	0.9500	C19—C20	1.376 (3)
C4—C5	1.404 (4)	C19—H19	0.9500
C5—C6	1.365 (4)	C20—H20	0.9500
C5—H5	0.9500	O1W—H1W	0.833 (10)
C6—H6	0.9500	O1W—H2W	0.831 (10)
C7—C8	1.463 (3)	O2W—H3W	0.839 (10)
C7—H7	0.9500	O2W—H4W	0.836 (10)
			
O2—N1—O1	121.7 (2)	C10—C9—C8	120.9 (3)
O2—N1—C1	119.1 (2)	C10—C9—H9	119.6
O1—N1—C1	119.3 (2)	C8—C9—H9	119.6
C4—N2—N3	119.5 (2)	C9—C10—C11	120.9 (2)
C4—N2—H2N	120.4	C9—C10—H10	119.5
N3—N2—H2N	120.1	C11—C10—H10	119.5
C7—N3—N2	115.9 (2)	C10—C11—C12	118.1 (2)
C14—N4—N5	116.7 (2)	C10—C11—C14	122.4 (2)
N4—N5—C15	119.9 (2)	C12—C11—C14	119.5 (2)
N4—N5—H5N	119.9	C13—C12—C11	121.0 (3)
C15—N5—H5N	120.2	C13—C12—H12	119.5
O3—N6—O4	121.8 (2)	C11—C12—H12	119.5
O3—N6—C18	118.8 (2)	C12—C13—C8	121.0 (2)
O4—N6—C18	119.5 (2)	C12—C13—H13	119.5
C6—C1—C2	121.1 (2)	C8—C13—H13	119.5
C6—C1—N1	119.0 (2)	N4—C14—C11	120.8 (2)
C2—C1—N1	119.9 (2)	N4—C14—H14	119.6
C3—C2—C1	119.2 (2)	C11—C14—H14	119.6
C3—C2—H2	120.4	N5—C15—C20	119.6 (2)
C1—C2—H2	120.4	N5—C15—C16	121.0 (2)
C2—C3—C4	120.2 (2)	C20—C15—C16	119.4 (2)
C2—C3—H3	119.9	C17—C16—C15	119.8 (2)
C4—C3—H3	119.9	C17—C16—H16	120.1
N2—C4—C3	119.3 (2)	C15—C16—H16	120.1
N2—C4—C5	121.4 (2)	C16—C17—C18	120.1 (2)
C3—C4—C5	119.3 (2)	C16—C17—H17	120.0
C6—C5—C4	120.3 (2)	C18—C17—H17	120.0
C6—C5—H5	119.9	C17—C18—C19	120.9 (2)
C4—C5—H5	119.9	C17—C18—N6	119.3 (2)
C5—C6—C1	119.9 (3)	C19—C18—N6	119.8 (2)
C5—C6—H6	120.0	C20—C19—C18	119.2 (2)
C1—C6—H6	120.0	C20—C19—H19	120.4
N3—C7—C8	120.8 (2)	C18—C19—H19	120.4
N3—C7—H7	119.6	C19—C20—C15	120.6 (2)
C8—C7—H7	119.6	C19—C20—H20	119.7
C13—C8—C9	118.1 (2)	C15—C20—H20	119.7
C13—C8—C7	122.8 (2)	H1W—O1W—H2W	117 (3)
C9—C8—C7	119.1 (2)	H3W—O2W—H4W	106 (3)
			
C4—N2—N3—C7	−176.2 (2)	C9—C10—C11—C14	−176.8 (2)
C14—N4—N5—C15	−179.5 (2)	C10—C11—C12—C13	−2.6 (4)
O2—N1—C1—C6	3.6 (3)	C14—C11—C12—C13	177.3 (3)
O1—N1—C1—C6	−177.0 (2)	C11—C12—C13—C8	0.3 (5)
O2—N1—C1—C2	−174.6 (2)	C9—C8—C13—C12	1.6 (4)
O1—N1—C1—C2	4.8 (3)	C7—C8—C13—C12	−177.9 (3)
C6—C1—C2—C3	−0.6 (4)	N5—N4—C14—C11	−178.8 (2)
N1—C1—C2—C3	177.6 (2)	C10—C11—C14—N4	−4.7 (4)
C1—C2—C3—C4	−0.5 (4)	C12—C11—C14—N4	175.3 (2)
N3—N2—C4—C3	179.1 (2)	N4—N5—C15—C20	−177.1 (2)
N3—N2—C4—C5	−1.1 (3)	N4—N5—C15—C16	3.0 (3)
C2—C3—C4—N2	−178.5 (2)	N5—C15—C16—C17	178.9 (2)
C2—C3—C4—C5	1.7 (4)	C20—C15—C16—C17	−0.9 (4)
N2—C4—C5—C6	178.3 (2)	C15—C16—C17—C18	1.2 (4)
C3—C4—C5—C6	−1.9 (4)	C16—C17—C18—C19	−0.3 (4)
C4—C5—C6—C1	0.8 (4)	C16—C17—C18—N6	−179.1 (2)
C2—C1—C6—C5	0.5 (4)	O3—N6—C18—C17	7.1 (3)
N1—C1—C6—C5	−177.7 (2)	O4—N6—C18—C17	−173.2 (2)
N2—N3—C7—C8	−179.8 (2)	O3—N6—C18—C19	−171.7 (2)
N3—C7—C8—C13	−2.3 (4)	O4—N6—C18—C19	7.9 (3)
N3—C7—C8—C9	178.2 (2)	C17—C18—C19—C20	−0.9 (4)
C13—C8—C9—C10	−1.1 (4)	N6—C18—C19—C20	177.9 (2)
C7—C8—C9—C10	178.4 (2)	C18—C19—C20—C15	1.2 (4)
C8—C9—C10—C11	−1.3 (4)	N5—C15—C20—C19	179.9 (2)
C9—C10—C11—C12	3.1 (4)	C16—C15—C20—C19	−0.3 (4)

### Hydrogen-bond geometry (Å, °)

**Table:**

*D*—H···*A*	*D*—H	H···*A*	*D*···*A*	*D*—H···*A*
O1W—H1w···O4^i^	0.833 (17)	2.32 (2)	3.084 (2)	153 (2)
O1W—H2w···O2w^ii^	0.83 (2)	2.01 (2)	2.808 (3)	163 (3)
N5—H5n···O1w	0.88	2.17	3.021 (3)	163
O2W—H3w···O1w^iii^	0.84 (3)	1.98 (3)	2.800 (3)	165 (3)
O2W—H4w···O1^iv^	0.84 (2)	2.28 (2)	3.061 (3)	156 (3)
O2W—H4w···O2^iv^	0.84 (2)	2.45 (2)	3.204 (3)	150 (3)
N2—H2n···O2w^v^	0.88	2.09	2.959 (3)	172
C7—H7···O2^vi^	0.95	2.48	3.374 (3)	157
C14—H14···O3^i^	0.95	2.45	3.338 (3)	156

Symmetry codes: (i) *x*−1, *y*−1, *z*; (ii) −*x*+1, −*y*+1, −*z*+1;  (iii) *x*, *y*+1, *z*; (iv) −*x*, −*y*, −*z*+2; (v) −*x*+1, −*y*+1, −*z*+2; (vi) *x*+1, *y*+1, *z*.
